# Supplementary material for: NCF4 attenuates colorectal cancer progression by modulating inflammasome activation and immune surveillance
Source: Nat Commun. 2024 Jun 17;15:5170. doi: 10.1038/s41467-024-49549-7 (PMC11183137; doi:10.1038/s41467-024-49549-7)
Supplement: Supplementary file 1 — Supplementary Information [file 41467_2024_49549_MOESM1_ESM.pdf]

# NCF4 attenuates colorectal cancer progression by modulating inflammasome activation and immune surveillance

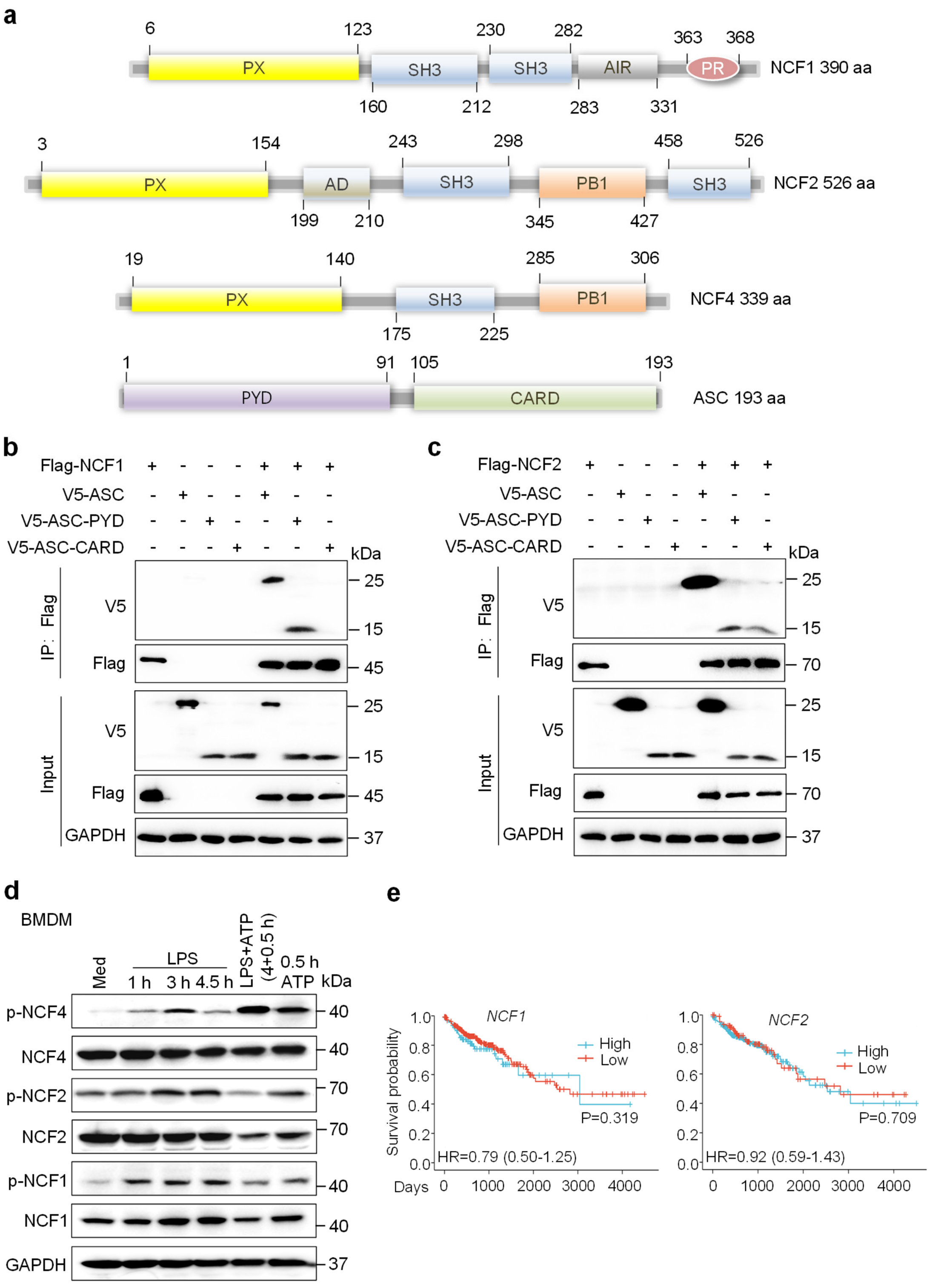


**Supplementary Figure 1. NCF1 and NCF2 interact with ASC.**

**a**, Schematic representation of full-length NCF1, NCF2, NCF4, and ASC with functional domains. PX domain is shared among NCF1, NCF2, and NCF4. PB1 domain is shared by NCF2 and NCF4.

**b**, Immunoblot analysis of FLAG-NCF1 co-IP with V5-ASC, V5-ASC-PYD, and V5-ASC-CARD from lysates of HEK293T cells transfected with the indicated plasmids.
**c**, Immunoblot analysis of FLAG-NCF2 co-IP with V5-ASC, V5-ASC-PYD, and V5-ASC-CARD from lysates of HEK293T cells transfected with the indicated plasmids.
**d**, Immunoblot analysis of total and phosphorylated NCF4, NCF1, and NCF2 in WT BMDMs treated with LPS alone (500 ng/mL), LPS (500 ng/mL) and ATP (5 mM), or ATP alone (5 mM) for the indicated times.
**e**, Correlation analysis between the gene expression of *NCF1* or *NCF2* and survival rate of CRC patients. (*NCF1*_High, n=99; *NCF1*_low, n=379; *NCF2*_High, n=363; *NCF2*_Low, n=115)
Data are representative of 3 independent experiments with similar results (**b**-**d**). Log-rank (Mantel-Cox) test for (**e**), *p* value is indicated in the graph. Source data are provided as a Source Data file.

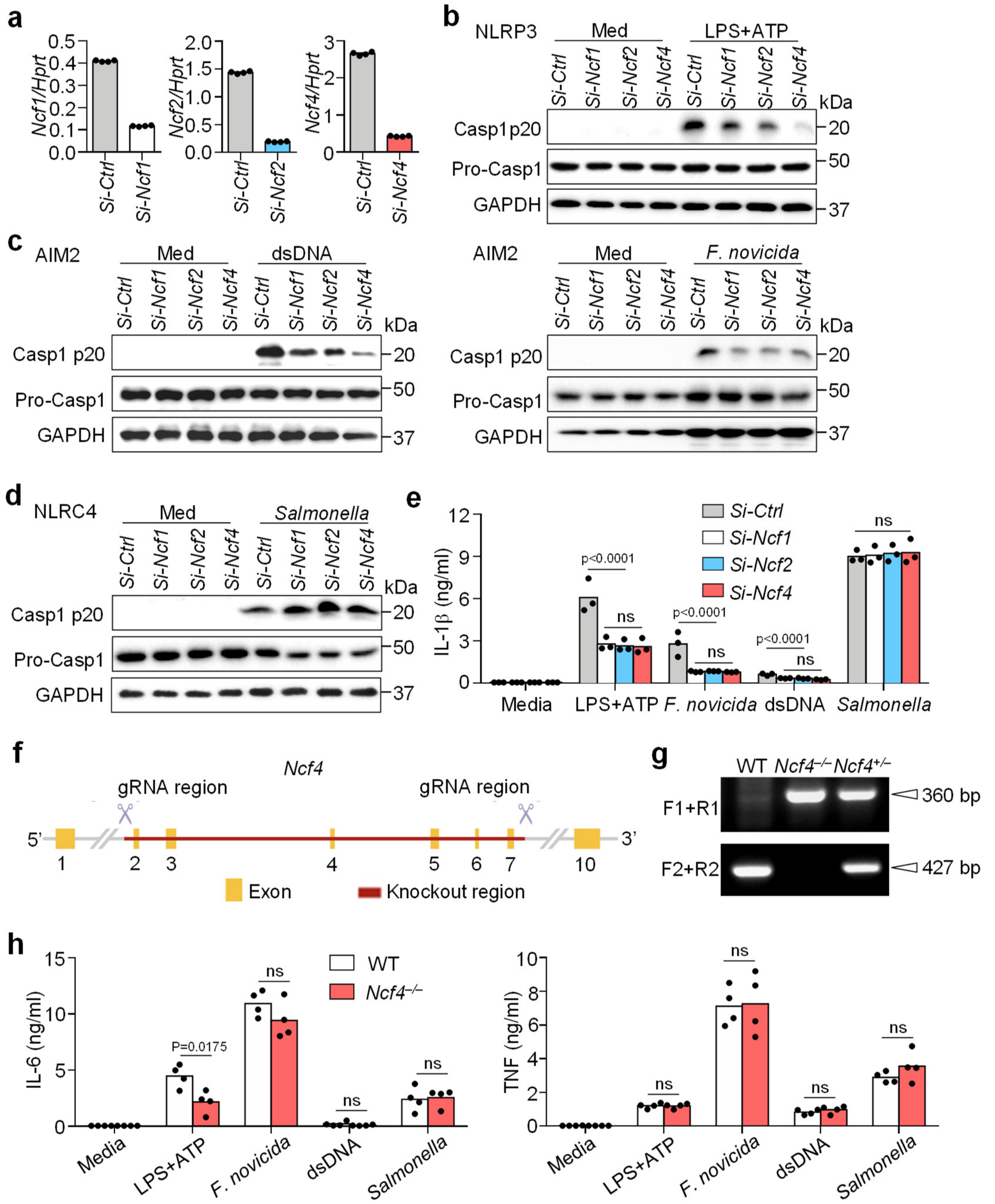


**Supplementary Figure 2. Knock-down of *Ncf1*, *Ncf2*, or *Ncf4* reduces the activation of the NLRP3 and AIM2 inflammasomes.**

**a**, qRT-PCR analysis of *Ncf1*, *Ncf2*, and *Ncf4* in WT BMDMs transfected with siRNA control and siRNAs specific to *Ncf1*, *Ncf2*, and *Ncf4* genes, respectively (n=4 technical replicates; 3 independent experiments).

**b**-**d**, Immunoblot analysis of pro-caspase-1 (Pro-Casp1) and its subunit p20 in WT BMDMs transfected with siRNAs in (**a**) and further treated with LPS (500 ng/mL, 4 h) and ATP (5 mM, 60 min) for NLRP3 inflammasome activation (**b**), transfected with dsDNA (1.5 μg, 2 h) or infected with *F. novicida* (200 MOI, 24 h) for AIM2 inflammasome activation (**c**), and *Salmonella enterica* Typhimurium (3 MOI, 4 h) for NLRC4 inflammasome activation (**d**).

**e**, Analysis of IL-1β release in WT BMDMs transfected with siRNAs and further treated with inflammasome activation stimuli in (**b**-**d**, n=3 biologically independent samples).
**f**,**g**, Targeting strategy used to generate *Ncf4*$^{-/-}$ mice (**f**) and genotyping of offspring generated from breeding of *Ncf4* heterozygous mice (**g**).
**h**, Analysis of IL-6 and TNF release in WT and *Ncf4*$^{-/-}$ BMDMs without treatment (Media) or stimulated with LPS (500 ng/mL, 4 h) and ATP (5 mM, 60 min), transfected with dsDNA (1.5 μg, 2 h), infected with *F. novicida* (200 MOI, 24 h), and *Salmonella enterica* Typhimurium (3 MOI, 4 h) (n=4 biologically independent samples).
Data are from 3 (**e**,**h**) or representative of 3 independent experiments with similar results (**a-d**). Data represent Mean±SEM for (**a, e, h**), 2-sided Student's t test without multiple-comparisons correction, *p* value is indicated in the graph. Source data are provided as a Source Data file.

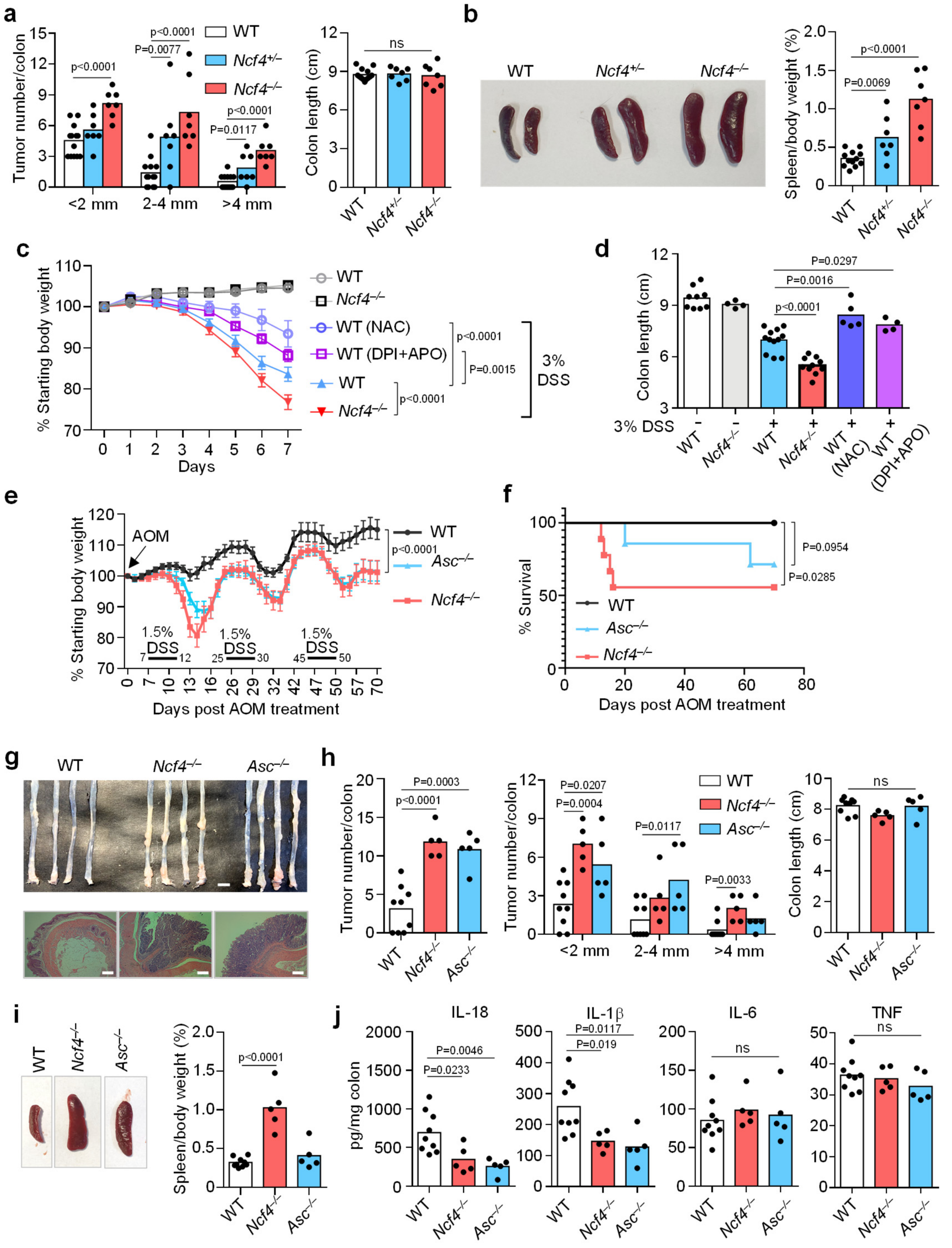

**Supplementary Figure 3. NCF4 and ASC prevent tumorigenesis of CRC.**

**a**, Colorectal tumor analysis in WT (n=13), *Ncf4*$^{+/-}$ (n=7), and *Ncf4*$^{-/-}$ (n=7) mice after AOM-DSS treatment.

**b**, Image and size of spleens from WT (n=12), *Ncf4*$^{+/-}$ (n=7), and *Ncf4*$^{-/-}$ (n=7) mice after AOM-DSS treatment.

**c**,**d**, Body weight change and colon length analysis of control and 3% DSS-treated gender- and age-matched WT and *Ncf4*$^{-/-}$ mice with or without ROS inhibitors as indicated. NAC (100 mg/kg), DPI (20 ng/kg), and APO (2 mg/kg)

were injected twice at day 2 and day 4. (WT, n=9; *Ncf4*$^{-/-}$, n=4; WT-NAC-DSS, n=5; WT-DPI/APO-DSS, n=4; WT-DSS, n=12; *Ncf4*$^{-/-}$-DSS, n=10)

**e**,**f**, Body weight change (**e**) and survival analysis (**f**) of gender- and age-matched WT (n=9), *Asc*$^{-/-}$ (n=7), and *Ncf4*$^{-/-}$ (n=9) mice after AOM injection at Day 0 and three rounds treatment of DSS as indicated.

**g**,**h**, Colorectal tumors in WT (n=9), *Asc*$^{-/-}$ (n=5), and *Ncf4*$^{-/-}$ (n=5) mice at day 70 under AOM-DSS treatment, and H&E staining of Colorectal tumors. Scale bars: 10 mm for upper panel and 10 μm for lower panel.

**i**, Image and size of spleens from WT (n=9), *Asc*$^{-/-}$ (n=5), and *Ncf4*$^{-/-}$ (n=5) mice after AOM-DSS treatment.

**j**, ELISA analysis of IL-18, IL-1β, IL-6, and TNF in colon tissues from WT(n=9), *Asc*$^{-/-}$ (n=5), and *Ncf4*$^{-/-}$ (n=5) mice in (**g**).

Data are from 2 (**c**-**j**) or representative of 3 independent experiments with similar results (**a**, **b**). Data represent Mean±SEM for (**a**, **b**, **d**, **h**-**j**), 2-sided Student's t test without multiple-comparisons correction, two-way ANOVA for (**e**), Log-rank (Mantel-Cox) test for (**f**), *p* value is indicated in the graph. Source data are provided as a Source Data file.

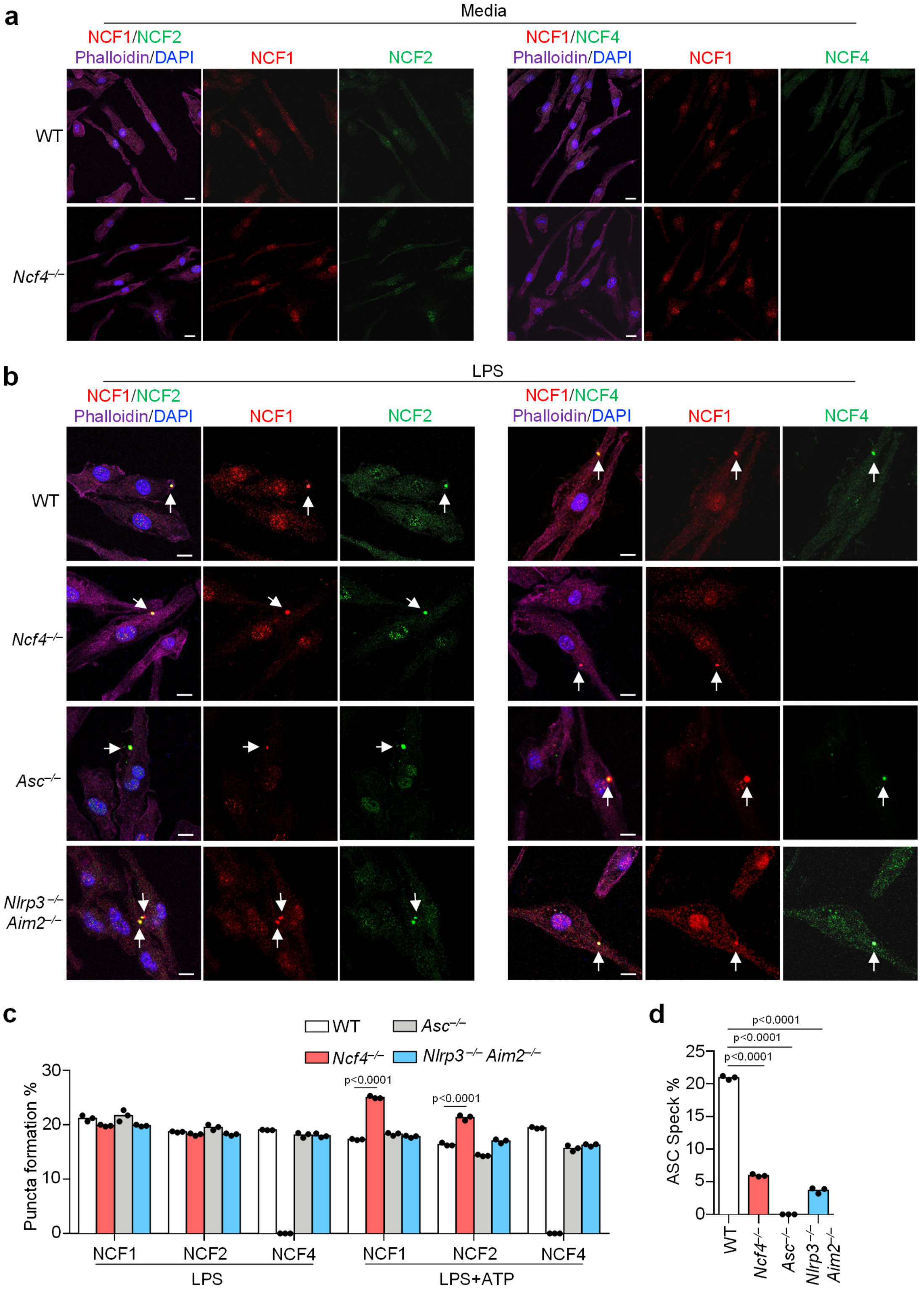


**Supplementary Figure 4. NCF4, NCF1, and NCF2 form a complex following LPS stimulation.**

**a**, Confocal microscopy analysis of co-localization of NCF1 and NCF2, NCF1 and NCF4 in untreated WT and *Ncf4*$^{-/-}$ BMDMs.

**b**, Confocal microscopy analysis of co-localization of NCF1 and NCF2, NCF1 and NCF4 in WT, *Ncf4*$^{-/-}$, *Asc*$^{-/-}$, and *Nlrp3*$^{-/-}$*Aim2*$^{-/-}$ BMDMs stimulated with

LPS (500 ng/mL, 4.5 h). Arrows indicate co-localized puncta. Scale bars: 10 μm.
**c**, Quantification analysis of the puncta formation of NCF1, NCF2, and NCF4 in WT, *Ncf4*$^{-/-}$, *Asc*$^{-/-}$, and *Nlrp3*$^{-/-}$*Aim2*$^{-/-}$ BMDMs stimulated with LPS alone or LPS plus ATP for NLRP3 inflammasome activation. At least 130 (130-200) cells were analyzed for each group (n=3 biologically independent samples).
**d**, Quantification of ASC speck formation in WT, *Ncf4*$^{-/-}$, *Asc*$^{-/-}$, and *Nlrp3*$^{-/-}$*Aim2*$^{-/-}$ BMDMs stimulated with LPS plus ATP for inflammasome activation. At least 130 (130-200) cells were analyzed for each group (n=3 biologically independent samples).
Data are from 3 (**c**, **d**) or representative of 3 independent experiments with similar results (**a**, **b**). Data represent Mean±SEM for (**c**, **d**), 2-sided Student's t test without multiple-comparisons correction, *p* value is indicated in the graph. Source data are provided as a Source Data file.

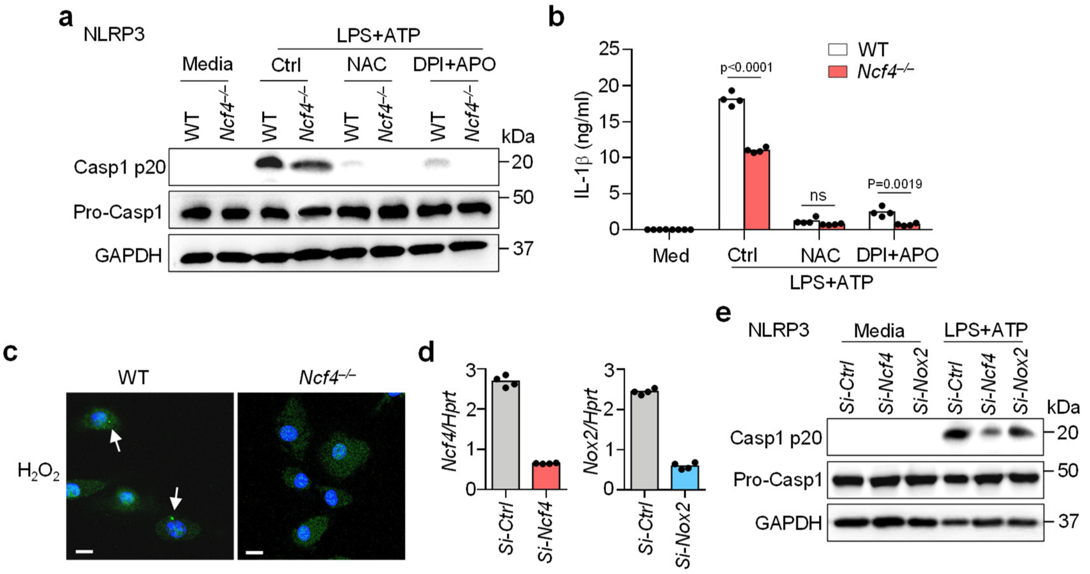


**Supplementary Figure 5. NOX2 is not essential for inflammasome activation.**

**a**,**b**, Immunoblot analysis of caspase-1 maturation (**a**) and ELISA analysis of IL-1β production (**b**, n=4 biologically independent samples) in control DMSO-treated (Ctrl) or ROS inhibitor-treated (NAC 25 μM and DPI/APO 20 μM/10 μM, 1 h) WT and *Ncf4*$^{-/-}$ BMDMs stimulated with LPS (500 ng/mL, 4 h) and ATP (5 mM, 60 min) for NLRP3 inflammasome activation.

**c**, Confocal microscopy analysis of ASC speck formation in WT and *Ncf4*$^{-/-}$ BMDMs stimulated with $H_2O_2$ (10 μM, 1 h). Scale bars: 10 μm.

**d**, Gene expression analysis of *Ncf4* and *Nox2* in WT BMDMs transfected with control siRNA and siRNAs specific to *Ncf4* and *Nox2* (n=4 technical replicates; 3 independent experiments).

**e**, Immunoblot analysis of caspase-1 maturation in *Si-Ctrl*, *Si-Ncf4*, and *Si-Nox2* transfected BMDMs further stimulated with LPS (500 ng/mL, 4 h) and ATP (5 mM, 60 min) for NLRP3 inflammasome activation.

Data are from 3 (**b**) or representative of 3 independent experiments with similar results (**a**, **c**-**e**). Data represent Mean±SEM for (**b**, **d**), 2-sided Student's t test without multiple-comparisons correction, *p* value is indicated in the graph. Source data are provided as a Source Data file.

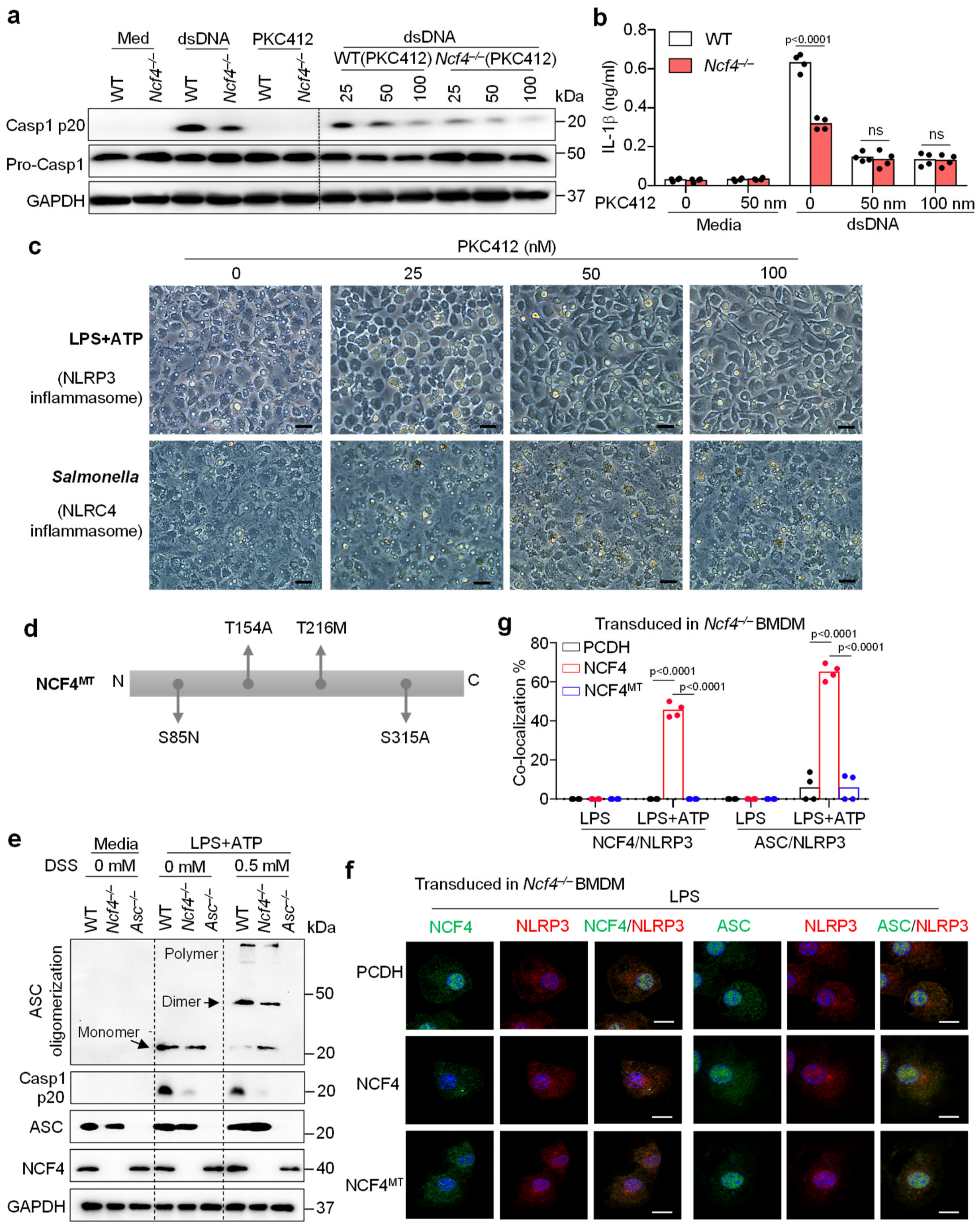

**Supplementary Figure 6. The phosphorylation of NCF4 is important for inflammasome activation.**

**a**,**b**, Immunoblot analysis of pro-caspase-1 (Pro-Casp1) and its subunit p20 (**a**), and analysis of IL-1β release (**b**, n=4 biologically independent samples) in PKC412-treated (25 nm, 50 nm and 100 nm for 1 hour) and untreated WT and *Ncf4*$^{-/-}$ BMDMs further transfected with dsDNA (1.5 μg, 2 h) for AIM2 inflammasome activation.

**c**, Microscopy analysis of cell death in untreated and PKC412-treated WT BMDMs, treated with either LPS plus ATP (LPS, 500 ng/mL, 4 h and ATP, 5 mM, 60 min) or *Salmonella enterica* Typhimurium (3 MOI, 2 h). Scale bars: 30 μm.

**d**, Schematic representation of phosphorylation site mutations of NCF4.
**e**, ASC oligomerization analysis in WT, *Asc*$^{-/-}$, and *Ncf4*$^{-/-}$ BMDMs without treatment (Media) or treated with LPS plus ATP (LPS, 500 ng/mL, 4 h and ATP, 5 mM, 30 min).
**f**, Confocal microscopy analysis NCF4, NCF4$^{MT}$, NLRP3, and ASC subcellular localization in *Ncf4*$^{-/-}$ BMDMs transduced with a control plasmid (PCDH), WT NCF4 and quadruple mutations (NCF4$^{MT}$) of phosphorylation sites of NCF4 response to LPS (500 ng/mL, 4.5 h). Scale bars: 20 μm.
**g**, Quantification analysis of the co-localization of NCF4, NCF4$^{MT}$, NLRP3, and ASC subcellular localization in *Ncf4*$^{-/-}$ BMDMs transduced with control plasmid (PCDH), WT NCF4 and quadruple mutations (NCF4$^{MT}$) of phosphorylation sites of NCF4 response to treatments with LPS (500 ng/mL, 4.5 h) or LPS plus ATP (LPS, 500 ng/mL, 4 h and ATP, 5 mM, 30 min). At least 200 cells were analyzed for each group (n=4 biologically independent samples).
Data are from 3 (**b**, **g**) or representative of 3 independent experiments with similar results (**a**, **c**-**f**). Data represent Mean±SEM for (**b**, **g**), 2-sided Student's t test without multiple-comparisons correction, *p* value is indicated in the graph. Source data are provided as a Source Data file.

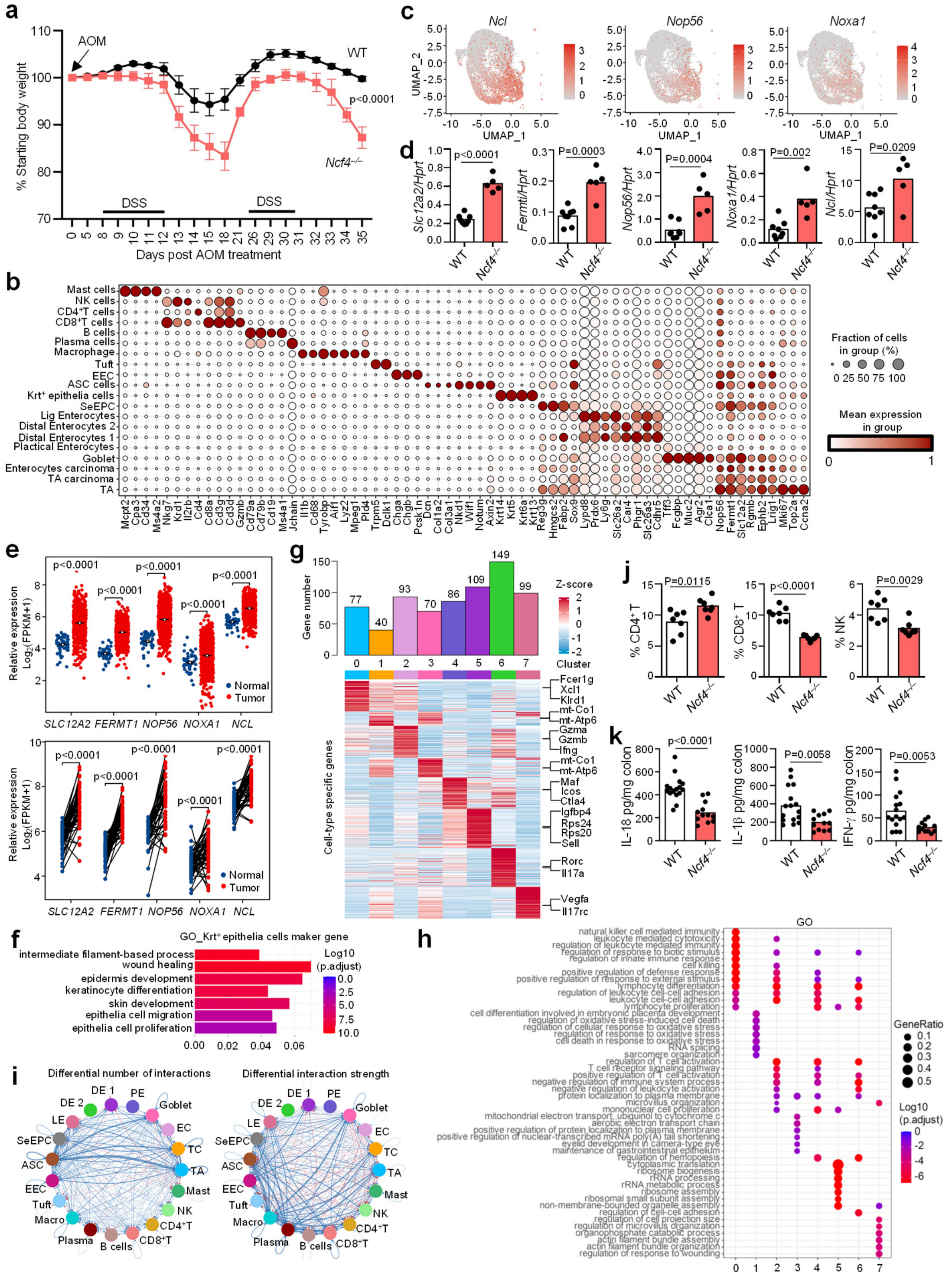


**Supplementary Figure 7. Single-cell RNA sequencing analysis of the cell composition in the colon tissues of WT and *Ncf4*–/– mice.**

Colonic tissues from gender- and age-matched WT and *Ncf4*–/– mice at Day 35 post AOM treatment were collected for single-cell RNA sequencing analysis.

**a**, Body weight change of WT (n=6) and *Ncf4*–/– mice (n=6) after AOM injection at Day 0 and two rounds treatment of DSS (2%) as indicated.

**b**, Bubble plot showing the expression of the selected marker genes for each cluster in single-cell RNA sequencing analysis of WT (n=3) and *Ncf4*–/– mice

(n=3).
**c**, UMAP plot of epithelial cells showing the expression of genes with stem-like features (tumor marker), *Ncl*, *Nop56*, and *Noxa1*.
**d**, Gene expression analysis of *Slc12a2*, *Fermt1*, *Ncl*, *Nop56*, and *Noxa1* in colonic tissues from WT (n=8) and *Ncf4*$^{-/-}$ (n=5) mice in (**a**).
**e**, Gene expression analysis of *SLC12A2*, *FERMT1*, *NCL*, *NOP56*, and *NOXA1* in total (upper) and paired (lower, n=41) colorectal tumors (n=480) and control tissues (n=41) from 480 colorectal cancer (CRC) patients in TCGA database.
**f**, GO analysis of the specifically expressed genes in Krt$^+$ epithelial cells.
**g**, Bar plot and Heatmap showing the number and the expression (z-score normalized) of cell-type specific genes for the sub-clusters of CD4$^+$ T, CD8$^+$ T, and NK cells.
**h**, GO analysis of cell-type specific genes for the sub-clusters of CD4$^+$ T, CD8$^+$ T, and NK cells.
**i**, Circle plot highlighting the different number and strength of ligand-receptor (L-R) interactions between WT and *Ncf4*$^{-/-}$ mice. The blue (red) line indicated the interaction was weaker (stronger) or less (more) in *Ncf4*$^{-/-}$ than WT mice. The line width represented amplitude of difference.
**j**, Quantification analysis of colonic CD4$^+$ T, CD8$^+$ T and NK cells from WT (n=7) and *Ncf4*$^{-/-}$ (n=7) mice as indicated.
**k**, ELISA analysis of IL-18, IL-1β, and IFN-γ production in colonic tissues from WT (n=16) and *Ncf4*$^{-/-}$ (n=12) mice as indicated.
Data are from 2 (**k**) or representative of 3 independent experiments with similar results (**a**, **d**, **j**). Data represent Mean±SEM for (**d**, **j**, **k**), 2-sided Student's t test without multiple-comparisons correction, two-way ANOVA for (**a**), Wilcoxon signed rank test for (**e**), *p* value is indicated in the graph. Source data are provided as a Source Data file.

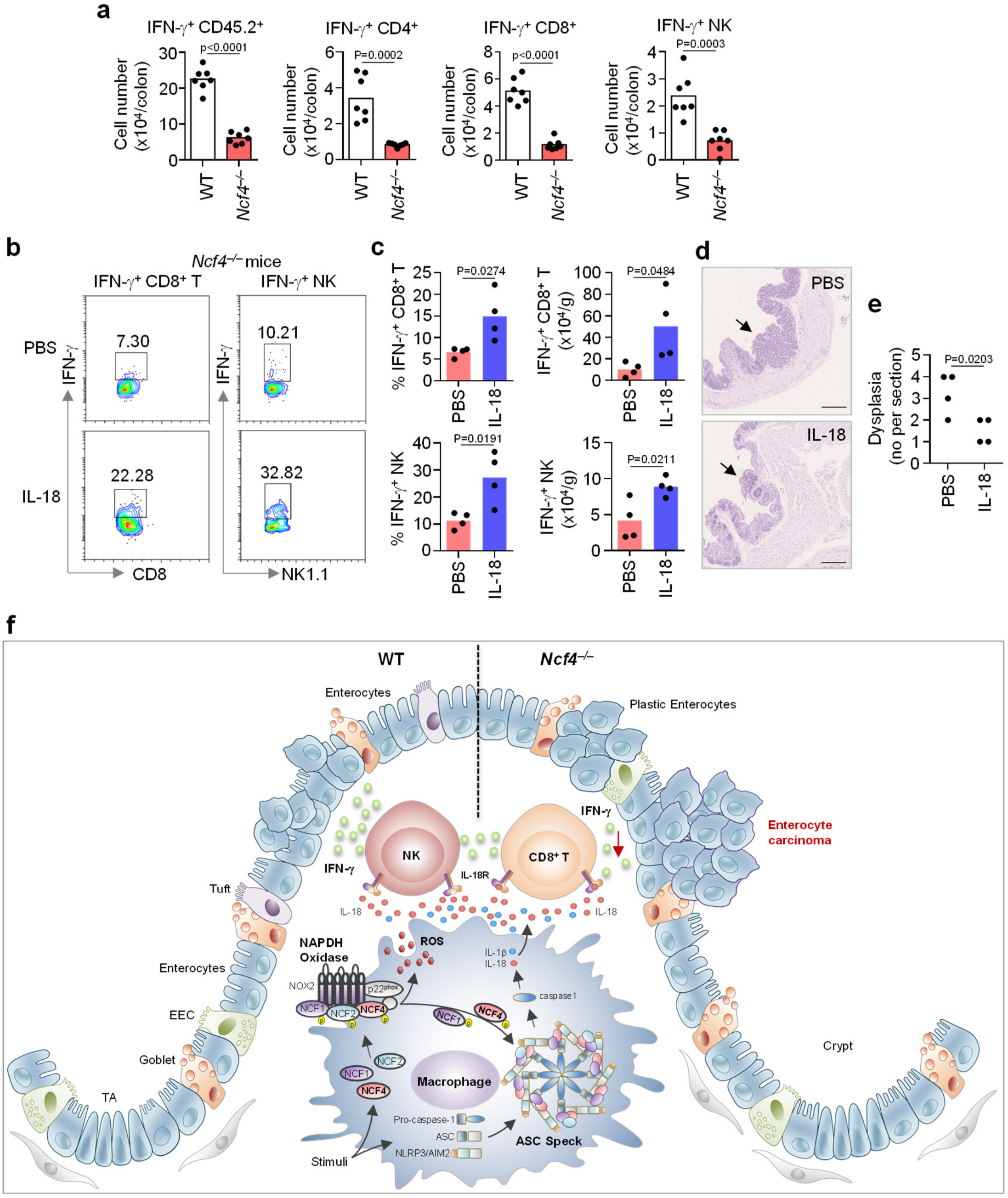


**Supplementary Figure 8. IL-18 injection promotes the activation of CD8$^{+}$ T cells and NK cells in *Ncf4*$^{-/-}$ mice with AOM-DSS treatment.**

**a**, Quantification analysis of the absolute number of IFN-γ$^{+}$ cells in colonic CD45.2$^{+}$ cells, CD4$^{+}$ T cells, CD8$^{+}$ T cells, and NK cells from WT (n=7) and *Ncf4*$^{-/-}$ (n=7) mice as indicated.

**b**, Representative flow cytometry plots of colonic IFN-γ$^{+}$ CD8$^{+}$ T cells and IFN-γ$^{+}$ NK cells from gender- and age-matched *Ncf4*$^{-/-}$ mice injected with IL-18 (n=4) or PBS (n=4) at Day 35 post-AOM treatment and treated with 1.5% DSS for two rounds as indicated in Extended Data Fig. 7a. At days 7, 9 ,11, 25, 27, and 29, mice were intraperitoneally injected with PBS or recombinant mouse IL-18 (5μg per mouse) six times.

**c**, Quantification analysis of the percentage and absolute number of IFN-γ$^{+}$

CD8$^{+}$ T cells and IFN-$\gamma^{+}$ NK cells from *Ncf4*$^{-/-}$ mice as in (**b**) (n=4 per group).
**d**, H&E staining of Colon tissues of *Ncf4*$^{-/-}$ mice injected with IL-18 (n=4) or PBS (n=4) at day 35 post-AOM-DSS treatment in (**b**). Arrows indicate epithelial dysplasia. Scale bars: 50 μm.
**e**, Quantification analysis of the number of epithelial dysplasia per transverse section in (**d**) (n=4 per group).
**f**, A model of NCF4 regulating inflammasome activation and inhibiting colorectal cancer development. Upon stimulation, NCF4 interacts with NCF1 and NCF2 to form a membrane-bound NADPH oxidase complex for ROS production. Following increased ROS signaling, NCF4 predominantly switches from the plasma membrane to the perinuclear region, and mediates ASC speck formation and inflammasome activation. In contrast, NCF4 deficiency leads to an impaired inflammasome-IFN-γ axis during the earlier phase of colorectal tumorigenesis, a reduction in the activation and recruitment of CD8$^{+}$ T cells and NK cells, and an increase in transit-amplifying (TA) and precancerous cells. EEC, enteroendocrine cells. Red arrow indicates reduced IFN-γ signaling in *Ncf4*$^{-/-}$ mice. Figure created by authors and artist Qing Li.
Data are from 2 (**b**-**e**) or representative of 3 independent experiments with similar results (**a**). Data represent Mean±SEM for (**a**, **c**, **e**) data, 2-sided Student's t test without multiple-comparisons correction, *p* value is indicated in the graph. Source data are provided as a Source Data file.

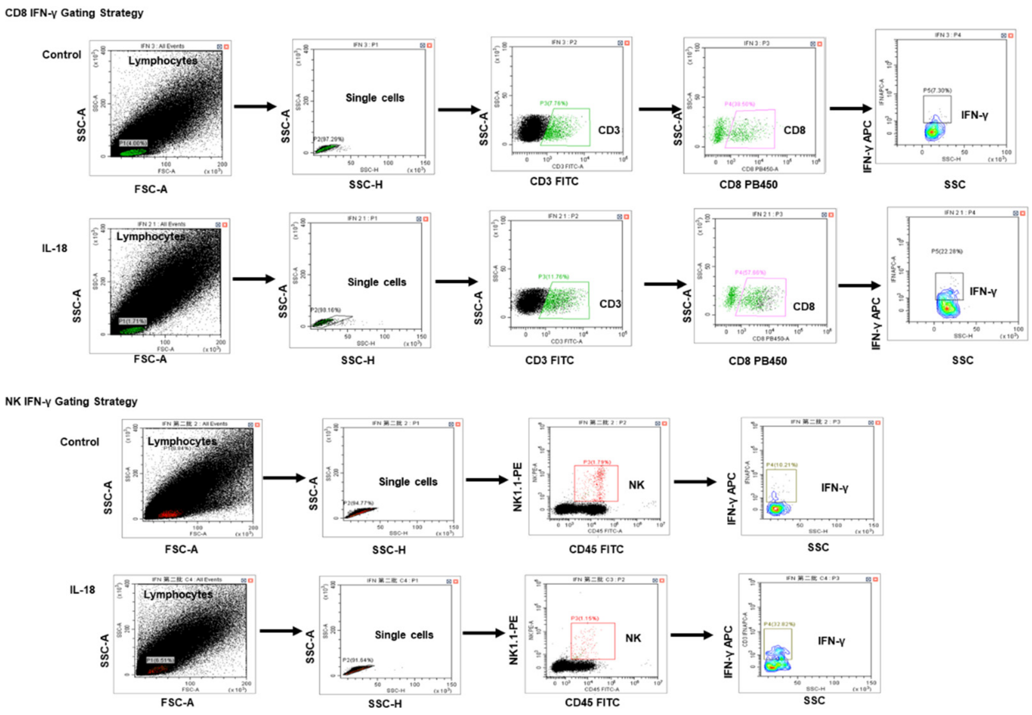


**Supplementary Figure 9. Gating strategies for Supplementary Figure 8b.**

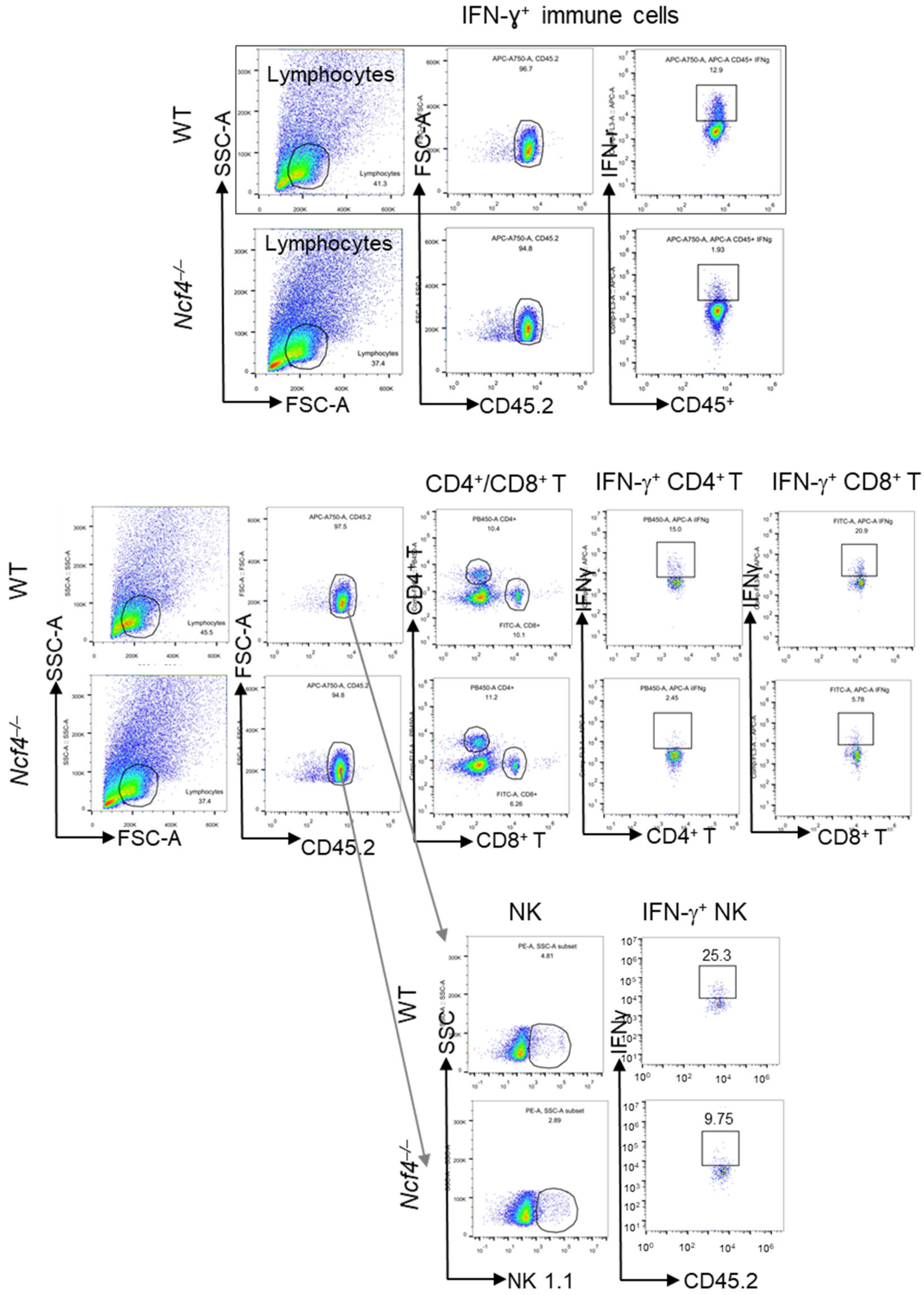


**Supplementary Figure 10. Gating strategies for Figure 7h.**

# Supplementary Table 1. Sequence of oligos used in this manuscript.

| **Gene** | **5' primer** | **3'primer** | |
|---|---|---|---|
| **qRT-PCR** | | | |
| *Hprt* | CTCATGGACTGATTATGGACAGGAC | GCAGGTCAGCAAAGAACTTATAGCC | |
| *Ncf1* | ACCTGTCGGAGAAGGTGGT | TAGGTCTGAAGGATGATGGG | |
| *Ncf2* | AGGACTATCTGGGCAAGGC | GCTGCGACTGAGGGTGAAT | |
| *Ncf4* | CTAAGTTTCAAAGCTGGAGATGTG | GTCCTCGGGAAAGTCCTTAAG | |
| *Nox2* | TCCTATGTTCCTGATCCTTTGTG | GTCCCACCTCCATCTTGAATC | |
| **Vector construction** | | | |
| Asc | ATATgcggccgcATGGGGCGGGCACGAGATGC | ATATgaattcTCAGCTCTGCTCCAGGTCCATC | |
| mNCF4 | ATATgaattcATGGCCCTGGCCCAGCAG | ATATctcgagTCAGGGGACAGTGTTGTAGACAC | |
| mNCF4-PX | ATATgaattcATGGCCCTGGCCCAGCAG | ATATctcgagTCACTGCTCAGCATCGTATGCAG | |
| mNCF4-SH3 | ATATgaattcATGGTGCCCCAGGCACTC | ATATctcgagTCAGTCCTCGTCCTCGGGAAAG | |
| mNCF4-PB1 | ATATgaattcATGACCACCAACTGGCTACGATG | ATATctcgagTCAGGGGACAGTGTTGTAGACAC | |
| mNCF4T85N | CTTTCACCTGCAATCTGCCCAC | GTGGGCAGATTGCAGGTGAAAG | |
| mNCF4T216M | CAGGGAGCCATGGGCATCTTCC | GGAAGATGCCCATGGCTCCCTG | |
| mNCF4T154A | GACCGCGCGCGCGCAAAATCAA | TTGATTTTGCGCGCGCGCGGTC | |
| mNCF4S315A | GCCTCCCTGCCCAGAAGC | GCTTCTGGGCAGGGAGGC | |
| mNCF1 | ATATgaattcATGGGGGACACCTTCATTCGCC | ATATctcgagTCACACAGCGGACGTCAGCTTC | |
| mNCF2 | ATATgaattcATGTCCCTGGCTGAGGCCATC | ATATctcgagCTAGACTTCTCTGGGAATGCCTTCC | |
| mASC-PYD | ATATggatccATGGGGCGGGCACGAGATGCC | ATATctcgagGGCTGAGCAGGGACACTGGCT | |
| mASC-CARD | ATATggatccATGTCTGGAGCTGTGGCAGCT | ATATctcgagGGGCTCTGCTCCAGGTCCATC | |
| **siRNAs sequence** | | | |
| si-m-*Ncf1* | CCAAAGATGGCAAGAATAA | CCATTGAGGTCATTCATAA | GCAGGTGAACCGTATGTAA |
| si-m-*Ncf2* | GGAACCAGCTGATAGACTA | CGTGAACACTATCCTGGAA | CCAAGGTCTTATTGATGAA |
| si-m-*Ncf4* | CCCTCAATGCCTACATGAA | AGCAAGAACAGCCCTTTCA | GTTCCAGAGAGAAGACATT |
| si-m-*Nox2* (Cybb) | GCTGAATGTCTTCCTCTTT | CCATGGAGCTGAACGAATT | GCACCATGATGAGGAGAAA |
| | | | |
