# Supplementary material for: NCF4 attenuates colorectal cancer progression by modulating inflammasome activation and immune surveillance
Source: Nat Commun. 2024 Jun 17;15:5170. doi: 10.1038/s41467-024-49549-7 (PMC11183137; doi:10.1038/s41467-024-49549-7)
Supplement: Supplementary file 3 — Description of Additional Supplementary Files [file 41467_2024_49549_MOESM3_ESM.pdf]

Description of Additional Supplementary Files

Supplementary Data 1. Co-IP analysis of ASC-interacting proteins. Mass spectrometry analysis of the IP product from the immunoprecipitated products generated with immunoprecipitation with an ASC antibody from the lysates of WT and *Asc*$^{-/-}$ BMDMs infected with *F. novicida* (100 MOI) for 12 hours. The proteins detected in WT but not *Asc*$^{-/-}$ BMDMs were listed. Exp1 and Exp2 indicate 2 independent experiments.

Supplementary Data 2. The list of cell type-specific genes for all cell clusters.
The marker genes were generated using the FindAllMarkers function in the Seurat R package. The gene name, cell type, expression change, percentage of cells with detected expression and the p value were included in the gene list. The Wilcoxon rank-sum tests with two.sided were used to calculate the p values.

Supplementary Data 3. The list of cell type-specific genes for subtypes of CD4^{+} T cells, CD8^{+} T cells, and NK cells.
The marker genes were generated using the FindAllMarkers function in the Seurat R package. The gene name, cell type, expression change, percentage of cells with detected expression and the p value were included in the gene list. The Wilcoxon rank-sum tests with two.sided were used to calculate the p values.

Supplementary Movie 1.
3D-Confocal microscopy movie analysis of co-localization of NCF1 and NCF2, NCF1 and NCF4 in WT BMDMs stimulated with LPS (500 ng/mL, 4.5 h), or in WT, *Ncf4*$^{-/-}$ and *Asc*$^{-/-}$ BMDMs stimulated with LPS (500 ng/mL, 4 h) and ATP (5 mM, 30 min) for NLRP3 inflammasome activation.
