# Supplementary material for: NCF4 attenuates colorectal cancer progression by modulating inflammasome activation and immune surveillance
Source: Nat Commun. 2024 Jun 17;15:5170. doi: 10.1038/s41467-024-49549-7 (PMC11183137; doi:10.1038/s41467-024-49549-7)
Supplement: Supplementary file 8 — Reporting Summary [file 41467_2024_49549_MOESM8_ESM.pdf]

nature portfolio

Corresponding author(s): Si Ming Man, Xiangbo Meng Tao Xu, Xiaopeng Qi

Last updated by author(s): May 29, 2024

Reporting Summary

Nature Portfolio wishes to improve the reproducibility of the work that we publish. This form provides structure for consistency and transparency in reporting. For further information on Nature Portfolio policies, see our Editorial Policies and the Editorial Policy Checklist.

Statistics

For all statistical analyses, confirm that the following items are present in the figure legend, table legend, main text, or Methods section.

n/a	Confirmed	
☐	☒	The exact sample size (*n*) for each experimental group/condition, given as a discrete number and unit of measurement
☐	☒	A statement on whether measurements were taken from distinct samples or whether the same sample was measured repeatedly
☐	☒	The statistical test(s) used AND whether they are one- or two-sided<br>*Only common tests should be described solely by name; describe more complex techniques in the Methods section.*
☒	☐	A description of all covariates tested
☐	☒	A description of any assumptions or corrections, such as tests of normality and adjustment for multiple comparisons
☐	☒	A full description of the statistical parameters including central tendency (e.g. means) or other basic estimates (e.g. regression coefficient) AND variation (e.g. standard deviation) or associated estimates of uncertainty (e.g. confidence intervals)
☐	☒	For null hypothesis testing, the test statistic (e.g. *F*, *t*, *r*) with confidence intervals, effect sizes, degrees of freedom and *P* value noted<br>*Give P values as exact values whenever suitable.*
☒	☐	For Bayesian analysis, information on the choice of priors and Markov chain Monte Carlo settings
☒	☐	For hierarchical and complex designs, identification of the appropriate level for tests and full reporting of outcomes
☒	☐	Estimates of effect sizes (e.g. Cohen's *d*, Pearson's *r*), indicating how they were calculated

Our web collection on statistics for biologists contains articles on many of the points above.

Software and code

Policy information about availability of computer code

Data collection	ZEISS-LSM880 and ANDOR High speed confocal microscope, ZEISS image acquisition, IMARIS 3-D construction were used data collection. The single cell RNA-seq experiments of mouse colon in WT and Ncf4–/– groups were performed using the Single Cell 3' RNA Reagent Kits (10x Genomics, Pleasanton, California) according to the manufacturer’s instruction.
Data analysis	Data analysis were performed using ZEN black_2-3SP1, ZEN blue 2.6, and IMARIS software. Graphpad Prism v 8, 9 for statistical data-analysis. The raw scRNA-seq data were firstly mapping to the mouse genome (mm10) and quantified using 10X Genomics Cellranger (v4.0.0). Then, the filtered feature-barcode matrix including features, barcode list and matrix was generated and regarded as input to Seurat (v4.3.0) . After removing the low-quality cells, the package DoubletFinder (v2.0.3) was used for doublet removal. After dimensionality reduction, the 'FindClusters' module in Seurat was employed to generate clusters. The cellular clusters were subsequently manually categorized into distinct cell types based on their marker gene expression. To achieve an equal count of total cells between WT and Ncf4–/– groups, the Ncf4–/– group underwent down sampling of cells. Cell-cell communication was analyzed and inferred using CellChat (v1.6.1) with the default parameters. To calculate the RNA velocity of the single cells, the CellRanger output BAM files were firstly transformed to loom files using velocyto CLI, and then calculated and visualized using scVelo. For more detailed information, please see the Method section in the manuscript.

For manuscripts utilizing custom algorithms or software that are central to the research but not yet described in published literature, software must be made available to editors and reviewers. We strongly encourage code deposition in a community repository (e.g. GitHub). See the Nature Portfolio guidelines for submitting code & software for further information.

Data

Policy information about availability of data

All manuscripts must include a data availability statement. This statement should provide the following information, where applicable:

- Accession codes, unique identifiers, or web links for publicly available datasets
- A description of any restrictions on data availability
- For clinical datasets or third party data, please ensure that the statement adheres to our policy

The single-cell RNA-seq datasets generated in this study have been deposited at GSA under accession number CRA010882 (https://ngdc.cncb.ac.cn/gsa/search?searchTerm=CRA010882). The proteomics datasets generated in this study are available via ProteomeXchange with identifier PXD052497 (http://www.ebi.ac.uk/pride/archive/projects/PXD052497).Source data are provided with this paper. The remaining data are available within the Article, Supplementary Information or Source Data file.

Research involving human participants, their data, or biological material

Policy information about studies with human participants or human data. See also policy information about sex, gender (identity/presentation), and sexual orientation and race, ethnicity and racism.

Reporting on sex and gender	*Use the terms sex (biological attribute) and gender (shaped by social and cultural circumstances) carefully in order to avoid confusing both terms. Indicate if findings apply to only one sex or gender; describe whether sex and gender were considered in study design; whether sex and/or gender was determined based on self-reporting or assigned and methods used.*<br>*Provide in the source data disaggregated sex and gender data, where this information has been collected, and if consent has been obtained for sharing of individual-level data; provide overall numbers in this Reporting Summary. Please state if this information has not been collected.*<br>*Report sex- and gender-based analyses where performed, justify reasons for lack of sex- and gender-based analysis.*
Reporting on race, ethnicity, or other socially relevant groupings	*Please specify the socially constructed or socially relevant categorization variable(s) used in your manuscript and explain why they were used. Please note that such variables should not be used as proxies for other socially constructed/relevant variables (for example, race or ethnicity should not be used as a proxy for socioeconomic status).*<br>*Provide clear definitions of the relevant terms used, how they were provided (by the participants/respondents, the researchers, or third parties), and the method(s) used to classify people into the different categories (e.g. self-report, census or administrative data, social media data, etc.)*<br>*Please provide details about how you controlled for confounding variables in your analyses.*
Population characteristics	*Describe the covariate-relevant population characteristics of the human research participants (e.g. age, genotypic information, past and current diagnosis and treatment categories). If you filled out the behavioural & social sciences study design questions and have nothing to add here, write "See above."*
Recruitment	*Describe how participants were recruited. Outline any potential self-selection bias or other biases that may be present and how these are likely to impact results.*
Ethics oversight	*Identify the organization(s) that approved the study protocol.*

Note that full information on the approval of the study protocol must also be provided in the manuscript.

Field-specific reporting

Please select the one below that is the best fit for your research. If you are not sure, read the appropriate sections before making your selection.

☒ Life sciences ☐ Behavioural & social sciences ☐ Ecological, evolutionary & environmental sciences

For a reference copy of the document with all sections, see nature.com/documents/nr-reporting-summary-flat.pdf

Life sciences study design

All studies must disclose on these points even when the disclosure is negative.

Sample size	No statistical methods were used to pre-determine sample sizes; In exploratory experiments a sample size of 3 to 6 animals per group was used, which was sufficient to detect relevant difference; typically we performed the experiments as three or more biologically independent repeats to ensure reproducibility.
Data exclusions	No data were excluded from analysis.
Replication	All experiments were repeated at least 2 times or more as indicated in each figure legend. Experimental findings were reliably reproduced between experiments.
Randomization	Mice of similar age and sex were used for all experiments.

Blinding	No blinding was performed.

Reporting for specific materials, systems and methods

We require information from authors about some types of materials, experimental systems and methods used in many studies. Here, indicate whether each material, system or method listed is relevant to your study. If you are not sure if a list item applies to your research, read the appropriate section before selecting a response.

Materials & experimental systems

n/a	Involved in the study	
☐	☒	Antibodies
☐	☒	Eukaryotic cell lines
☒	☐	Palaeontology and archaeology
☐	☒	Animals and other organisms
☒	☐	Clinical data
☒	☐	Dual use research of concern
☒	☐	Plants

Methods

n/a	Involved in the study	
☒	☐	ChIP-seq
☐	☒	Flow cytometry
☒	☐	MRI-based neuroimaging

Antibodies

Antibodies used	The following primary antibodies were used: anti-ASC (AdipoGen, AG-25B-0006); anti-V5 (Cell Signaling Technology [CST], 13202); anti-caspase-1 (AdipoGen, AG-20B-0042); anti-NCF4 (Abcam, ab76158); anti-NCF1 (Abcam, ab166930); anti-NCF2 (Abcam, ab109366); anti-p-NCF4 (CST, 4311); anti-p-NCF1 (Affinity, AF3167); anti-p-NCF2 (Affinity, AF4343); anti-NOX2 (Abcam, ab129068); anti-FLAG (Sigma, F3165); anti-Myc (CST, 2278); anti-GAPDH (CST, 5174); anti-NKCC1 (Abcam, ab303518) and anti-KINDLIN (Abcam, ab68041). HRP-labeled anti-rabbit, anti-mouse or anti-goat (CST) was used as the secondary antibody.
Validation	All antibodies were valigated based on the manufacturer information available for each reference cited.

Eukaryotic cell lines

Policy information about cell lines and Sex and Gender in Research

Cell line source(s)	HEK-293T cells were purchased from Conservation Genetics Chinese Academy of Sciences Kunming Cell Bank.
Authentication	We purchase cell lines from the company, record passages, test for mycoplasma, clearly mark, and frequently assess markers. According to documents from company, they have been authenticated by STR analysis.
Mycoplasma contamination	Yes, any mycoplasma contamination has been excluded.
Commonly misidentified lines (See ICLAC register)	Not listed.

Animals and other research organisms

Policy information about studies involving animals; ARRIVE guidelines recommended for reporting animal research, and Sex and Gender in Research

Laboratory animals	Ncf4–/– mice were generated by Cyagen Biosciences Inc. Exons 2 through 7 of the Ncf4 gene were knocked out by CRISPR-Cas9 system. ApcMin/+ mice were purchased from Cyagen Biosciences Inc. Aim2–/– mice were provided by F. Shao (National Institute of Biological Sciences, Beijing, China), and Nlrp3–/– mice were provided by D. Wang (Zhejiang University, Hangzhou, China).WT and knockout mice were kept under specific pathogen-free conditions in the Animal Resource Center at Shandong University, Jinan, Shandong Province, China.
Wild animals	No wild animals were used in this study.
Reporting on sex	Male and Female mice were used for all experiments.
Field-collected samples	No field collected samples were used in this study.
Ethics oversight	All animal experiments were conducted in accordance with guidelines approved by the Ethics Committee of Scientific Research of Shandong University.

Note that full information on the approval of the study protocol must also be provided in the manuscript.

Plants

Seed stocks

Report on the source of all seed stocks or other plant material used. If applicable, state the seed stock centre and catalogue number. If plant specimens were collected from the field, describe the collection location, date and sampling procedures.

Novel plant genotypes

Describe the methods by which all novel plant genotypes were produced. This includes those generated by transgenic approaches, gene editing, chemical/radiation-based mutagenesis and hybridization. For transgenic lines, describe the transformation method, the number of independent lines analyzed and the generation upon which experiments were performed. For gene-edited lines, describe the editor used, the endogenous sequence targeted for editing, the targeting guide RNA sequence (if applicable) and how the editor was applied.

Authentication

Describe any authentication procedures for each seed stock used or novel genotype generated. Describe any experiments used to assess the effect of a mutation and, where applicable, how potential secondary effects (e.g. second site T-DNA insertions, mosiacism, off-target gene editing) were examined.

Flow Cytometry

Plots

Confirm that:

- [x] The axis labels state the marker and fluorochrome used (e.g. CD4-FITC).
- [x] The axis scales are clearly visible. Include numbers along axes only for bottom left plot of group (a 'group' is an analysis of identical markers).
- [x] All plots are contour plots with outliers or pseudocolor plots.
- [x] A numerical value for number of cells or percentage (with statistics) is provided.

Methodology

Sample preparation

Cells were isolated from colon were lysed using RBC lysis buffer, filtered through a 70 um filter, and then immediately spun down in the centrifuge. Finally, cells were resuspended in FACS buffer and used for subsequent analysis.

Instrument

Data were collected and analyzed on cytoflex S (Beckman Coulter, Brea,CA , US).

Software

FlowJo v.10.7.

Cell population abundance

No cell sorting was performed.

Gating strategy

Gating based on FSC area vs width and SSC area. Gates were drawn as shown in therelevant panels or as described in Nature Immunology, 2018, 19: 342-353.

- [x] Tick this box to confirm that a figure exemplifying the gating strategy is provided in the Supplementary Information.
